# The Value of Time

**Published:** 1891-03

**Authors:** 


### The Value of Time.

A correspondent, writing from a large commercial city, informs us that he remembers seeing many years ago, the following in some mercantile offices: "Call upon a business man at business time only, and on business. Transact your business and go about your business, in order to give him time to finish his business." Our correspondent feelingly asks whether a notice similar to the above, with the necessary modifications, might not be hung up in the consulting room and surgery of every busy consultant or general practitioner. Much has been said as to the rapidity with which the out-patients of hospitals are disposed of. But this rapidity is perfectly compatible with correctness of diagnosis, prognosis, and prescribing by an experienced practitioner, and there is something to be said on the other side—the time wasted by patients in prolix descriptions and tedious repetitions. The quaint story of the lady who consulted Abernethy and, knowing his impatience of such verbosity, held out her wounded finger and answered in monosyllables, is well known, and the example might be followed with great advantage. The lady in question was rewarded by Abernethy's impromptu praise, that she was the most rational woman he had ever met in his life. Those patients who are most considerate for their doctor's time are certainly the most welcome.—*Lancet.*

For Soldering Aluminum.—

| | | | | | |
|---|---|---|---|---|---|
| Gold | 30 parts. | or, | Gold | 50 parts. | |
| Silver | 20 " | | Silver | 10 " | |
| Platina | 1 " | | Copper | 10 " | |
| Aluminum | 100 " | | Aluminum | 20 " | |

(Schlosser)

